# Public and patient involvement: a survey on knowledge, experience and opinions among researchers within a precision oncology European project

**DOI:** 10.1186/s12885-023-11262-x

**Published:** 2023-08-30

**Authors:** Paola Mosconi, Cinzia Colombo, Pasquale Paletta, Laura Gangeri, Chiara Pellegrini, Elena Garralda, Rosalba Miceli, Cinzia Brunelli, on behalf of CEE_DART Consortium, Irene Braña, Jordi Rodon, Guillermo Villacampa, Anna Pedrola, Rodrigo Dienstmann, Bianca Pont, Júlia Lostes, Alejandro Piris, Elena Chavarria, Xenia Villalobos, Berta Colldeforns, Raquel Pérez-López, Paolo Nuciforo, David Tamborero, Janne Lehtiö, Ali Razzak, Maria Pernemalm, Markus Jonsson, Maan Rachid, Jorrit Boekel, Luigi de Petris, Christina Von Gertten, Helena Bäckvall, Maria Von Witting, Xiaobing He, Richard Baird, Thomas Jaki, Duncan Jodrell, Gary Doherty, Simon Pacey, Rebecca Bradley, Ferida Gallagher, Ramona Woitek, Emma Beddowes, Shubha Anand, Katherine Honan, Haiyan Zheng, Pavel Mozgunov, Nikos Demetris, Kate Donoghue, Kenneth Seamon, Lorena Escudero, Melanie Burton, Otso Arponen, Stefan Fröhling, Richard Schlenk, Petra Oberrauch, Anett Molnar, Manuel Störzel, Klaus Maier-Hein, Oliver Sedlaczek, Heinz-Peter Schlemmer, Peter Horak, Marco Nolden, Simon Kreutzfeldt, Michael Schlander, Philipp Schader, Muchadeyi Muchandifung, Jennifer Wessely, Frans Opdam, Regina Beets-Tan, Zuhir Bodalal, Ruud Weijer, Giovanni Apolone, Giovanni Scoazec, Claudio Vernieri, Rita Leporati, Luca Agnelli, Andrea Vingiani, Mikol Antioco, Silvia Damian, Matteo Duca, Filippo De Braud, Andrea Villa, Sara Alfieri, Arnauld Forest, Laura Lauden, Marc Deloger, Yohan Loriot, Emma Pailler, Paul Fitzpatrick, André Freitas, Ciara Dwan, Donna Graham, Hannah Frost, Leanna Goodwin, Alex Bogatu, Oskar Wysocki, Magdalena Wysocka, Sjoerd van Hagen, Bas Leenknegt, Sander Rodenburg, Elena Garcia, Pim van Nierop, Mirko Orsini, Marco Monari, Marco Pacchioni, Emma Mescoli, Enrico Calanchi

**Affiliations:** 1 Laboratory of Medical Research and Consumer Involvement, Department of Medical Epidemiology, Istituto di Ricerche Farmacologiche Mario Negri IRCCS, Via Mario Negri 2, 20156 Milan, Milan, Italy; 2 Clinical Psychology Department, Fondazione IRCCS Istituto Nazionale dei Tumori, Milan, Italy; 3 Palliative Care, Pain Therapy and Rehabilitation Unit, Fondazione IRCCS Istituto Nazionale dei Tumori, Milan, Italy; 4 Vall d’Hebron Institute of Oncology (VHIO) and Vall d, Hebron University Hospital, Barcelona, Spain; 5 Department of Clinical Epidemiology and Trial Organization, Fondazione IRCCS Istituto Nazionale dei Tumori, Milan, Italy

**Keywords:** Patient and public involvement, Participatory research, Patient engagement, Precision oncology, Survey

## Abstract

**Background:**

Patient and Public Involvement (PPI) is slowly but steadily being implemented in all phases of clinical research. As part of the European project “Building Data Rich Clinical Trials” a survey was launched to investigate the knowledge, experiences and opinions on this topic of clinicians and researchers from seven European clinical and non-clinical centers (Cancer Core Europe).

**Methods:**

An invitation to take part in a cross-sectional web survey was sent to 199 clinicians and researchers working in the field of precision oncology. The questionnaire was developed ad hoc because no existing questionnaires met the purpose of this study. The analysis takes account of whether respondents had experience on PPI or not.

**Results:**

On a total of 101 respondents, this survey reveals that 76.2% of them knew about PPI before answering the questionnaire, 54.5% had experience in the previous five years and 86.1% were interested in a training course on this topic. PPI knowledge grew together with career seniority (peak of 86.5% for established career professionals), while the group most interested in a course was the early-career professionals (100.0%). Finally, the majority of respondents stated they had no training or education on PPI (67.3% of experienced and 82.6% of not-experienced respondents).

**Conclusions:**

This survey shows that most cancer researchers knew the term PPI, even if only a little more than half of them had any relative experience. Opinions on PPI benefits, negative effects, barriers and requirements differed between the groups of PPI experienced and not-experienced respondents, showing that experience itself can influence respondents’ opinions. Most of respondents reported they would prefer a training course based on practical rather than theoretical tools.

**Supplementary Information:**

The online version contains supplementary material available at 10.1186/s12885-023-11262-x.

## Background

Since the early 1990s the scientific medical literature has published continuous reports of experiences and coined new terminologies to define the action field of lay involvement in health debates [1–4]. A few years ago the editorial “Let the patient revolution begin” emphasized the importance for clinicians and patients of working in partnership to improve healthcare, research, clinical practices and behaviors [5]. With the concept of Patient and Public Involvement (PPI)—also referred to as Participatory Research—this partnership was extended to the whole research process, including the identification of research priorities [6–9]. PPI is generally defined as «research carried out “with” or “by” members of the public – patients, service users and/or carers, or patient representatives – rather than “to”, “about” or “for” them» [10]. PPI entails active collaboration among all research stakeholders – the seven identified by Concannon and colleagues as public and patients, health providers, purchasers, payers, policy makers, product makers and researchers/principal investigators [11]. The main areas of interest are the prioritization of research topics, selection of study design and outcomes, planning and conduction of the study, and dissemination of the research results [10]. However, some clinicians and researchers are concerned about the role, ability, and expertise of lay people as partners [12]. Currently PPI is considered a core component of valuable research practice [12, 13]. More and more often funders evaluate research projects on the ability to achieve full involvement of patients. When publishing the results, details about the PPI level adopted are also often required [14].

Oncology was one of the first medical settings where patients’ organizations actively required investments for research, assistance and prevention. The battles against breast cancer, followed by ovarian, colon and blood cancers, have progressively promoted dynamic partnerships with clinicians and researchers [15–17]. PPI is therefore the natural result of years of activism and participation in the cancer area, where research has given encouraging results.

A systematic review identified 27 PPI studies reporting experience in oncological settings. The results indicate that PPI frontrunners in cancer research are mainly the UK and the USA, followed by Australia, at least in terms of publications, and that the studies have increasingly considered PPI in the early stages of research even though some issues, such as the representativeness of lay people involved and the real impact of PPI on the research agenda, still need to be clarified [18]. Therefore PPI needs to be integrated more broadly into cancer research: strategies to really capture the patients’ perspectives, training and educational initiatives for health professionals, funding and conditions are all still needed [9, 14, 19–23].

The CCE_DART Project (Building Data Rich Clinical Trials – https://cce-dart.com/) is a project funded by the European Union and conducted within the Cancer Core Europe (CCE – https://cancercoreeurope.eu/). The project involves clinical as well as non-clinical centers across Europe and its aim is to develop new and more efficient methods for the design, conduct and analysis of clinical trials in the field of precision oncology. One specific work package is dedicated to promotion of the active involvement of patients and the public through patient empowerment, web tools for information and participation, and sharing experiences on clinical trials in precision oncology (iEnter, iConsent, and iParticipate, respectively). Another work package aims at developing a training course, particularly dedicated to clinicians and health researchers, to provide the fundamentals of PPI together with the tools to conduct PPI-based research in the best possible way.

Before the organization of this CCE_DART training course, a multicenter survey was designed to explore knowledge, experience and opinions about PPI among clinicians and research personnel – with particular attention to PPI experienced respondents (PPI-ER) and not-experienced respondents (PPI-nER) – and to investigate their preferences about a PPI-related training course.

## Methods

### Study design, target population and sample selection

An invitation to take part in a cross-sectional web survey was sent to a convenience sample of the CCE_DART consortium professionals involved in cancer research (identified by local principal investigators): medical doctors, nurses, biologists, psychologists, statisticians, physiotherapists, computer scientists, bio-informaticians, epidemiologists, informaticians, project managers, economists. The countries involved were France, Germany, Italy, Spain, Sweden, The Netherlands, and the United Kingdom.

### Questionnaire

An online questionnaire was developed starting from a literature review, and through a process involving researchers and clinicians. Eligible papers were those published from January 1990 to May 2021, using any research approach (qualitative, quantitative or mixed) and any study design (including systematic reviews and surveys). In this review we considered meaningful knowledge, opinions, attitudes and experiences of clinicians and researchers in involving patients, survivors and carers at any stage of the research process. The outcomes of interest were challenges, barriers and impact of PPI in relation to research phases and methods. More details regarding the literature review will be published elsewhere.

As the literature search revealed only a limited number of questionnaires, and none of them fully fitted our aims, we collected questions from selected articles and set out a comparative table according to three domains: knowledge, experience, and opinions on PPI. The questionnaire was built selecting relevant items from 21 articles identified by the review (Additional file 1).

The questionnaire was pilot-tested by three researchers not involved in the project, who were invited to evaluate the completeness of the questionnaire, the comprehensibility of the questions and the answer options, to identify relevant aspects not considered, and the time needed to complete the questionnaire. Some questions were re-formulated and layout changes were made on the basis of these suggestions.

The final version (Additional file 2) is composed of: i) a set of questions about respondents’ socio-demographic characteristics (sex, year of birth, education, professional role, seniority, research setting and time spent on research); ii) a formal definition of PPI, aimed at avoiding misunderstandings; and iii) questions about PPI knowledge, experience, opinions and training needs. Survey data were collected and managed using REDCap, Research Electronic Data Capture, an electronic data capture tool [24].

### Sample size

The survey was planned to involve professionals working in DART consortium centers (20 researchers from each clinical center and 10 from each non-clinical one), and reminders were sent to non-respondents until a response rate of at least 40% was reached (expected minimum sample size 76). It was calculated that this minimum sample size gives a 95% confidence interval (CI) for proportions with a precision (half of the CI width) of 11.2% in the hypothesis of maximum variability (proportion equal to 50%) [25].

### Data analysis

We considered as respondents all participants who answered the first three questions of the survey – in addition to the sociodemographic ones – dealing with the broader topics of interest (Additional file 2, questions number 01, 03 and 04). Basic descriptive statistics was applied to analyze the data. Exact confidence interval estimates (95% CI) were calculated for the proportions of YES answers to the three main questions about PPI: knowledge, experience and interest in training. Data were analyzed conducted using SAS 9.4 software (SAS Institute, Cary, NC, USA).

## Results

The survey was launched in December 2021. An initial sample of 177 professionals was invited to participate, later extended to 199 to facilitate data collection in some centers with a low response rates. In March 2022, five reminders had been sent before data collection was closed.

A total of 106 professionals started the survey; one did not give consent to participation and four answered an insufficient number of questions to be considered respondents, leaving 101 total respondents (50.8% response rate). The final number of respondents exceeded the minimum expected sample size of 76 and the 40% response rate. All the respondents completed the whole questionnaire (Additional file 3).

Table 1 shows personal information, with data on institutional role and activities of respondents. As shown by their ages (25–66 years) and career stage, they are well distributed among young and senior professionals, most of whom (61.4%) spent more than 50% of their time in research. At the professional level too, distribution was balanced: 32.7% were clinical staff (medical doctors and nurses), 37.6% non-clinical researchers (biologists, statisticians, computer scientists, etc.), and 29.7% other research staff (project managers, study coordinators, economists, etc.).

**Table 1:** Characteristics of the 101 respondents

	Overall	
	No	%
Sex		
Male	41	40.6
Female	60	59.4
Age^a^		
Mean (Range)	43.9	(25–66)
Education^a^		
PhD	47	47.0
Master's Degree	43	43.0
Bachelor's or less	8	8.0
Other	2	2.0
Professional level		
Non-clinical researchers	38	37.6
Research staff	30	29.7
MD	28	27.7
Nurse	5	5.0
Position^b^		
Clinician/researcher/nurse	45	45.9
Unit/Laboratory/Department Director	29	29.6
Project manager	12	12.2
Trainee (Research fellow, PhD candidate, student)	8	8.2
Administrative staff	4	4.1
Career stage		
Established career (16 + years)	37	36.6
Mid-career (6–15 years)	37	36.6
Early-career (≤ 5 years)	27	26.7
Primary research setting		
Hospital/Research hospital	52	51.5
Other research Institute	25	24.8
University	19	18.8
Small or medium-sized enterprise	5	5.0
Percentage of working time spent on research		
< 25%	23	22.8
26%-50%	16	15.8
> 50%	62	61.4
Priority level of PPI in cancer research by the Institution of respondents^a^		
Medium	35	35.0
Don’t know	27	27.0
High	19	19.0
Low	19	19.0

^a^1 non-respondent

^b^3 non-respondents

Table 2 shows respondents’ answers to the three main questions about PPI: knowledge, experience and interest in training. A large number of respondents said they had heard about PPI before the explanation provided in the survey (76.2%, 95% CI 66.7% to 85.1%), and around half (54.5%, 95% CI 44.2% to 64.4%) had experience in PPI with previous or current research. The majority of respondents (86.1%, 95% CI 77.8% to 92.2%) said they were interested in a training course on PPI. Respondents with an established career were more likely than others to know the expression PPI, the proportion decreases in line with career stage (respectively 86.5%, 78.4% and 59.3% for established, mid- and early-career researchers). This was not so noticeable for the frequency of experience in PPI, since established and early-career researchers gave similar percentages (respectively 62.2% and 59.3%), while mid-career researchers were less experienced (43.2%). Finally, interest in attending a course on PPI was very high for all three groups, reaching 100% in the early-stage career group.

**Table 2:** Respondents’ knowledge, experience and interest in a training course on PPI

	Have you ever heard the expression “public and patient involvement”, usually abbreviated to PPI?	Have you ever involved public (patients, service users and/or carers, or patient representatives) in your cancer research activity?	Would you be interested in participating in a PPI training course?
**OVERALL SAMPLE** (No. 101)	**No. (%)**	**No. (%)**	**No. (%)**
No	24 (23.8)	46 (45.5)	14 (13.9)
Yes	77 (76.2)	55 (54.5)	87 (86.1)
**CAREER STAGE**			
Established career (16 + years) (No. 37)			
No	5 (13.5)	14 (37.8)	6 (16.2)
Yes	32 (86.5)	23 (62.2)	31 (83.8)
Mid-career (6–15 years) (No. 37)			
No	8 (21.6)	21 (56.8)	8 (21.6)
Yes	29 (78.4)	16 (43.2)	29 (78.4)
Early career (≤ 5 years) (No. 27)			
No	11 (40.7)	11 (40.7)	0 (0.0)
Yes	16 (59.3)	16 (59.3)	27 (100.0)

The next sections illustrate the comparison between the 55 PPI-ER and 46 PPI-nER.

Most of PPI-ER (45, 81.8%) said they had included PPI in their cancer research projects in the last five years, and 67.3% (37) were satisfied with implementing PPI. The most frequent reasons for PPI inclusion in cancer research were to accomplish a moral and ethical duty (29, 52.7%), to achieve better results (29, 52.7%), and to fulfill funders’ requests (23, 41.8%).

PPI was mostly applied in protocol development (26, 47.3%), writing/reviewing information sheets (24, 43.6%), and establishing research priorities (21, 38.2%).

Finally, the lay people most involved were mainly patients and/or potential patients (44, 80%), and organizations representing patients (32, 58.2%).

**Fig. 1:**
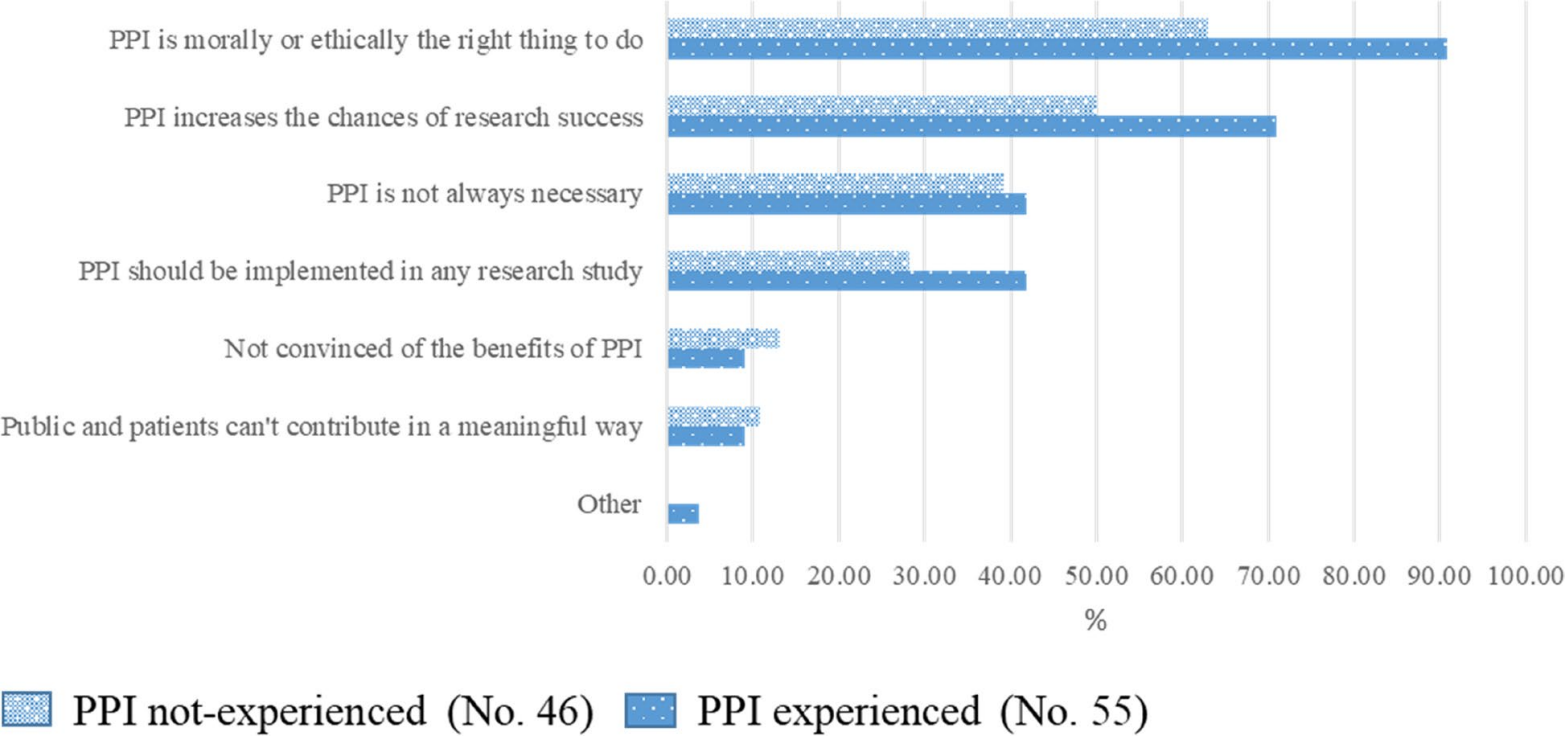
Opinions about PPI among experienced and not-experienced respondents. Legend: Respondents could select multiple items with no restrictions. The two bars corresponding to each item represent the percentage of respondents selecting that item out of the total of PPI not-experienced or PPI experienced, respectively

Opinions about PPI are shown in Fig. 1. For more than 90.9% of PPI-ER, PPI is morally/ethically the right thing to do, compared to 63.0% of PPI-nER. For more than 70.9% of PPI-ER, PPI increases the chances of success compared to 50.0% of PPI-nER. PPI-ER showed caution in considering PPI a research need, and deemed it not always necessary in oncology research (both 41.8%); these percentages were lower among PPI-nER (respectively 28.3% and 39.1%). Finally, only about 10.9% of PPI-ER and 9.1% of PPI-nER felt that patients could not contribute meaningfully to research and were not convinced about the benefits of PPI.

**Fig. 2:**
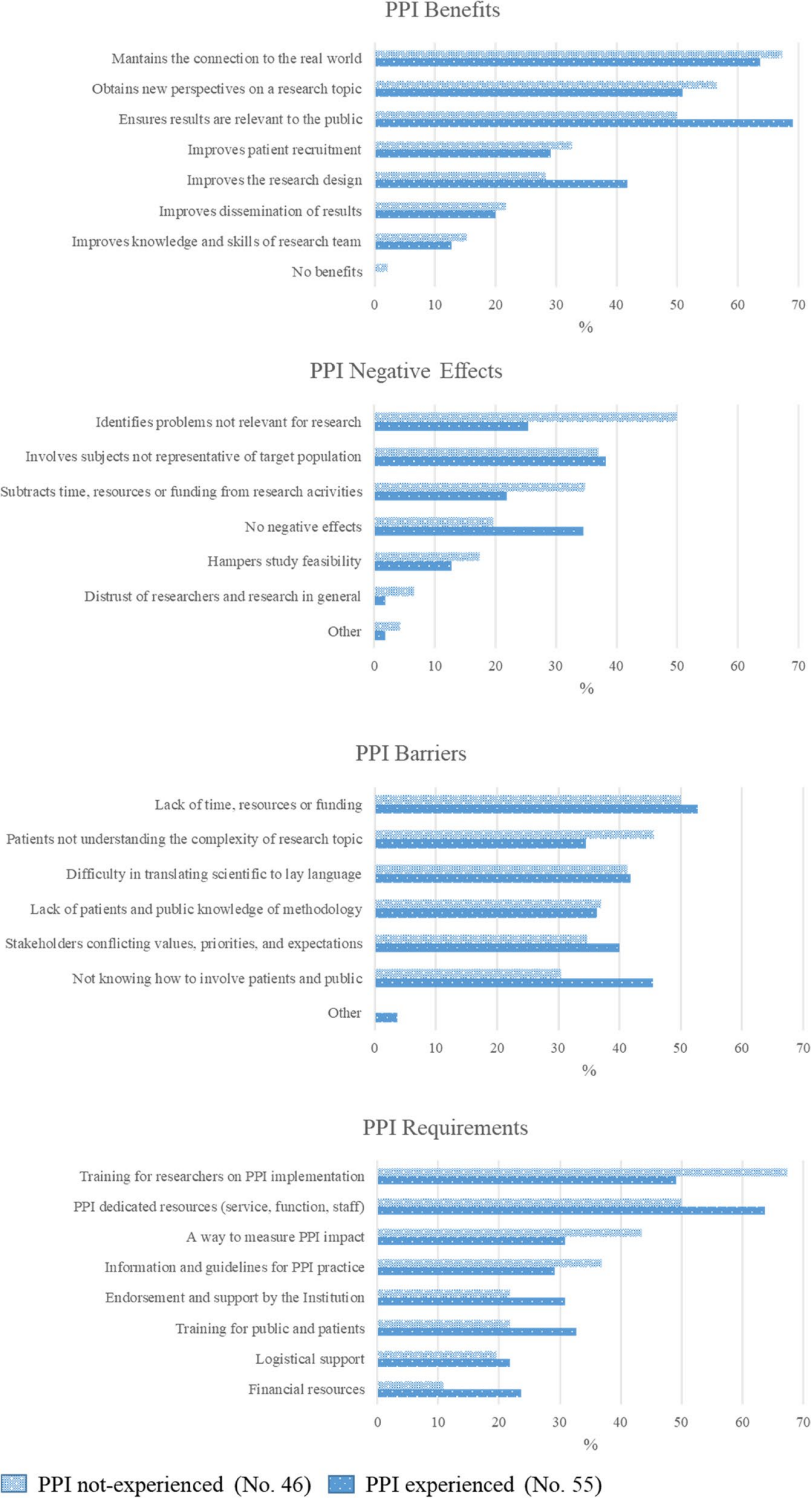
Benefits, negative effects, barriers and requirements about PPI among experienced and not-experienced respondents. Legend: Respondents could select multiple items with no restrictions on number. The two bars corresponding to each item represent the percentage of respondents selecting that item out of the total of PPI not-experienced or PPI experienced, respectively

Figure 2 shows respondents’ perceptions of benefits, negative effects, barriers and requirements of PPI. The most frequently perceived benefits of PPI-based research were the creation of a connection between research and the real world (63.6% PPI-ER versus 67.4% PPI-nER), results relevant to the public (69.1% PPI-ER versus 50.0% PPI-nER) and the identification of new perspectives on a research topic (50.9% PPI-ER versus 56.5% PPI-nER). Smaller numbers of respondents said there were no negative effects in implementing PPI in oncology research (34.6% PPI-ER versus 19.6% PPI-nER), while the most perceived negative effects were that PPI does not always involve people representative of the target population (around 37% for both respondent groups) and that it focuses on problems not relevant to research (25.5% PPI-ER versus 50.0% PPI-nER). The most frequent perceived barrier in PPI implementation (around 50% of both groups) was the lack of time, resources and funding. Among the other barriers, almost equally perceived (30–45% of both groups) was poor knowledge on how to involve patients, difficulties in communication with patients and conflicting priorities and expectations between researchers and patients. The most perceived PPI requirements were the need for training on PPI for researchers (67.4% PPI-nER) and for dedicated resources and funding (63.6% PPI-ER).

Respondents were also asked whether they would implement PPI in their research even if not required by the funders, and 63.6% of PPI-ER answered affirmatively versus 28.3% of PPI-nER. Regarding the possibility of offering payment to lay people involved in PPI, 29.1% of PPI-ER answered that it was important versus 17.4% of PPI-nER, while the importance of authorship of lay people in peer-reviewed publications was recognized by 56.4% of PPI-ER and 50.0% of PPI-nER.

**Fig. 3:**
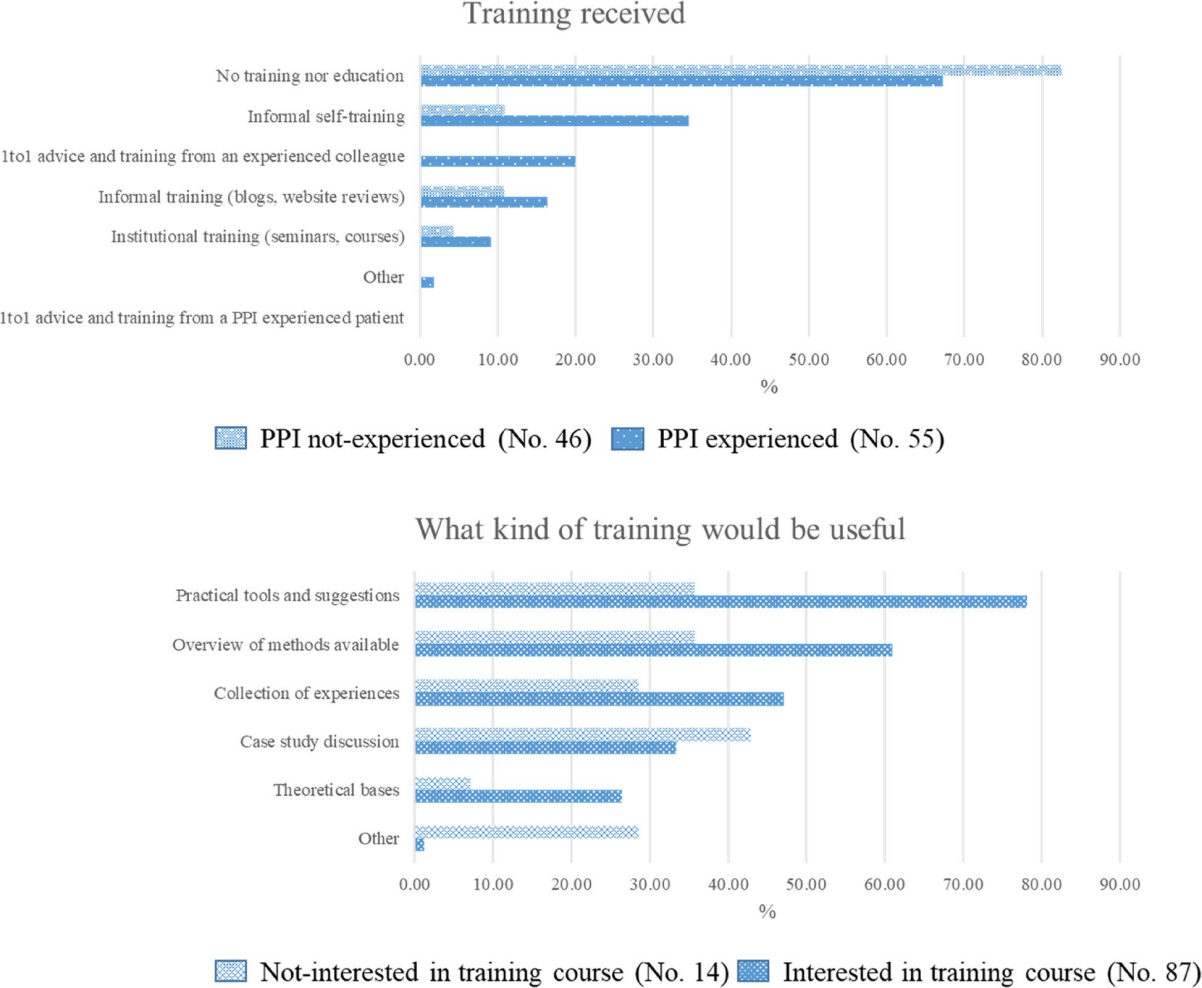
Actual training on PPI received and kind of training deemed useful. Legend: Respondents could select multiple items: up to three for section “Training received” and with no restrictions for the others. The two bars corresponding to each item represent the percentages of respondents selecting that item out of the total of PPI not-experienced or PPI experienced (top panel) or the total number of not interested and interested in training course, respectively (bottom panel)

Concerning training (Fig. 3), 67.3% of PPI-ER and 82.6% of PPI-nER said they had not received any formal training or education on PPI, while 9.1% of PPI-ER and 4.4% of PPI-nER said they had received institutional training. A large proportion of respondents (78.2%) interested in a training course would like to receive practical tools and suggestions on PPI (vs. 35.7% of not-interested), 60.9% wanted an overview on PPI methods available (vs. 35.7% of not-interested) and only 26.4% theoretical bases of PPI (vs. 7.1% of not-interested).

## Discussion

In the medical and scientific community PPI has been increasingly considered important to drive the research agenda according to the needs of both patients and clinicians, with the aim to increase the value, quality and integrity of research [26, 27]. This survey, involving seven European comprehensive cancer centers and four non-clinical centers, shows that the concept of PPI is known to most researchers; nevertheless, improvements are still needed to spread its principles and implement it more widely.

Overall, the term PPI has entered the respondents’ wordlist: two-thirds had heard of it, with a growing trend related to career seniority. PPI experience was inversely related to career stages, and in fact early career respondents had heard less about it. These findings suggest that the topic is not part of the educational path of the youngest health professionals. This is supported by the high levels of interest of the youngest respondents in a training course (Table 2). The need for adequate training on PPI is often stressed in the literature [22, 23, 26], as mentioned by researchers conducting clinical trials in a variety of settings [20].

Many respondents stated that an approach involving citizens, patients, caregivers and their representatives had important ethical and moral value (Fig. 1). It is also noticeable that some respondents considered PPI not always necessary or not always to be implemented. Even among PPI-ER there was some doubt that lay representatives could contribute meaningfully.

Researchers still have doubts about the feasibility of the involvement of lay components in research projects, but this topic is still more theoretical than practical [26, 28].

The implementation of PPI varies widely and is not adequately formalized in European healthcare systems and research settings [21]. Possible barriers are the lack of infrastructures, guidance and support [21]. Generally there are more perceived benefits than negative effects in implementing PPI in oncological research. Results relevant to the public are the most important benefit according to the PPI-ER, as noted in the literature [29]. Only a few PPI-nER insist there are no benefits with PPI.

Interestingly, one of the main negative effects of PPI perceived by both PPI-ER and PPI-nER is that lay subjects are considered not always representative of the target patient population, and this can undermine the representativeness of the results [18]. These concerns come from the observation that research teams may include subjects who are not fully representative of the clinical population under discussion (specifically for some characteristics, such as sex, education, ethnicity, or socio-economics factors) [18]. Furthermore, the PPI-nER seem more concerned than PPI-ER about the focus and the management of PPI conducted research. They consider that this involvement does not identify problems relevant to the research topic itself, and also that it subtracts time, resources and funds to research.

It is important to note that more than a third of PPI-ER think there are no negative effects in implementing PPI in cancer research, suggesting how experience is important for its fruitful application. Among the PPI-nER, measurement of PPI impact is also a critical requirement for effective PPI implementation in cancer research.

Grounding research on patients’ needs is fundamental in this field to improve clinical outcomes and their quality of life. Involving patients is therefore crucial, also considering the increasing production of studies on treatments whose value should be assessed in the light of the needs of people with cancer [30–33].

It is interesting that the results related to barriers to PPI implementation did not differ much between PPI-ER and PPI-nER. The lack of time, resources and funding is the aspect most frequently perceived as a barrier to PPI. Both groups have the same opinions about barriers to PPI implementation in cancer research, except for the lack of knowledge of methodology – which concerns the PPI-nER group more – and the scant understanding of the complexity of research topics by representatives of patients or the public – which concerns PPI-ER more. Independent training initiatives on research topics addressed to citizens, patients and their representatives should therefore be considered worth-while in order to overcome this perceived gap [34–36]. Citizen’s health literacy should also be taken into account – and probably improved – in order to encourage a more active and aware involvement. The European Union is carefully considering these aspects, and the CEE_DART Project devotes much attention to this as well, applying the PPI on a complex issue such as precision medicine.

Most respondents reported no training or education on PPI, one third of PPI-ER reported informal self-training, while only a few received structured or formal training. Training and dedicated resources in terms of service, function, and staff are in fact the most important requirements identified by respondents with and without PPI experience. As observed by Yu et al., as a result of a programme of 72 training workshops, attendees were more likely to involve patients in their research [22]. Providing early-stage researchers with appropriate educational, interactive, real-world training will arouse awareness of the merits and challenges associated with PPI [26]. This information will be useful to finalize one of the aims of the CCE_DART Project related to the design of a PPI training course targeted to researchers and health professionals.

This study investigated PPI in oncology research in a European setting, thus providing a new perspective compared to studies in other geographic areas and research fields.

Some peculiarities of this survey need to be underlined. After several reminders, 50.8% of the invited professionals responded to the survey, a response rate even better than literature reports [37, 38], considering, however, the narrow interest in the subject of the survey. The sample is heterogeneous in terms of professions, but is limited to a group of European research centers.

Since more than two-thirds of respondents spend half their working time on research they are very active interlocutors who could well benefit from wider implementation of the PPI. Considering the multidisciplinary approach as an added value for medical research, the results of this survey lend themselves to debate among a large number of stakeholders.

## Conclusions

To our knowledge, this is one of the earliest European surveys on these topics addressed to oncology researchers [18]. Interest in PPI is unquestionable as is the need for ad hoc training to boost its understanding and enhance skills for its implementation in research and practice. For meaningful implementation of PPI, systematic collection of experience and results is also needed. The CCE_DART Project, considering PPI a crucial element for two ad hoc designed working packages, is encouraging PPI development and implementation in the very challenging field of precision oncology research.

### Supplementary Information

**Additional file 1.** References used for questionnaire development.

**Additional file 2.** Questionnaire.

**Additional file 3.** Participants’ flowchart.

## Data Availability

The datasets used and/or analyzed for this study are available from the corresponding author on reasonable request.

## Abbreviations

CCE: Cancer Core Europe
CI: Confidence Interval
DART: Building Data Rich Clinical Trials
GDPR: General Data Protection Regulation
IRCCS: Istituto di Ricovero e Cura a Carattere Scientifico
PPI: Patient and Public Involvement
PPI-ER: PPI experienced respondents
PPI-nER: PPI not-experienced respondents
