# Supplementary material for: Public and patient involvement: a survey on knowledge, experience and opinions among researchers within a precision oncology European project
Source: BMC Cancer. 2023 Aug 30;23:814. doi: 10.1186/s12885-023-11262-x (PMC10470190; doi:10.1186/s12885-023-11262-x)
Supplement: Supplementary file 1 — Additional file 1. References used for questionnaire development. [file 12885_2023_11262_MOESM1_ESM.docx]

**Additional file 1.** References used for questionnaire development

(1) Chambers R, O'Brien LM, Linnell S, Sharp S. Why don't health researchers report consumer involvement? Quality in Primary Care 2004;12(2):151-157.

(2) Crocker JC, Boylan A, Bostock J, Locock L. Is it worth it? Patient and public views on the impact of their involvement in health research and its assessment: a UK‐based qualitative interview study. Health Expectations 2017;20(3):519-528.

(3) Crockett LK, Shimmin C, Wittmeier KD, Sibley KM. Engaging patients and the public in Health Research: experiences, perceptions and training needs among Manitoba health researchers. Research involvement and engagement 2019;5(1):1-11.

(4) de Wit M, Beurskens A, Piškur B, Stoffers E, Moser A. Preparing researchers for patient and public involvement in scientific research: development of a hands‐on learning approach through action research. Health Expectations 2018;21(4):752-763.

(5) Demagny L, Bungener M, Faurisson F. Engagement des chercheurs auprès des associations-Quel role jouent leurs opinions, leur activité clinique et leur genre? médecine/sciences 2015;31(11):1039-1045.

(6) Dudley L, Gamble C, Allam A, Bell P, Buck D, Goodare H, et al. A little more conversation please? Qualitative study of researchers’ and patients’ interview accounts of training for patient and public involvement in clinical trials. Trials 2015;16(1):1-15.

(7) Dudley L, Gamble C, Preston J, Buck D, EPIC Patient Advisory Group, Hanley B, et al. What difference does patient and public involvement make and what are its pathways to impact? Qualitative study of patients and researchers from a cohort of randomised clinical trials. PloS one 2015;10(6):e0128817.

(8) Ellis LE, Kass NE. Patient engagement in patient-centered outcomes research: challenges, facilitators and actions to strengthen the field. Journal of Comparative Effectiveness Research 2017;6(4):363-373.

(9) Gafos M, South A, Hanley B, Brodnicki E, Hodson M, McCormack S, et al. “PROUD to have been involved”: an evaluation of participant and community involvement in the PROUD HIV prevention trial. Research involvement and engagement 2020;6(1):1-13.

(10) Gelkopf MJ, Avramov I, Baddeliyanage R, Ristevski I, Johnson SA, Flegg K, et al. The Canadian retinoblastoma research advisory board: a framework for patient engagement. Research involvement and engagement 2020;6(1):1-21.

(11) Hawke LD, Darnay K, Brown M, Iyer S, Ben‐David S, Khaleghi‐Moghaddam M, et al. INNOVATE Research: Impact of a workshop to develop researcher capacity to engage youth in research. Health Expectations 2020;23(6):1441-1449.

(12) Hawke LD, Darnay K, Relihan J, Khaleghi‐Moghaddam M, Barbic S, Lachance L, et al. Enhancing researcher capacity to engage youth in research: researchers’ engagement experiences, barriers and capacity development priorities. Health Expectations 2020;23(3):584-592.

(13) Jørgensen CR, Eskildsen NB, Johnsen AT. User involvement in a Danish project on the empowerment of cancer patients–experiences and early recommendations for further practice. Research involvement and engagement 2018;4(1):1-13.

(14) Jose C, George-Zwicker P, Tardif L, Bouma A, Pugsley D, Pugsley L, et al. “We are the stakeholders with the most at stake”: scientific and autism community co-researchers reflect on their collaborative experience in the CONNECT project. Research involvement and engagement 2020;6(1):1-15.

(15) Kaisler RE, Missbach B. Co-creating a patient and public involvement and engagement ‘how to’guide for researchers. Research involvement and engagement 2020;6(1):1-10.

(16) Kapiriri L. Stakeholder involvement in health research priority setting in low income countries: the case of Zambia. Research involvement and engagement 2018;4(1):1-9.

(17) Kaur N, Pluye P. Delineating and operationalizing the definition of patient-oriented research: a modified e-Delphi study. Journal of Patient-Centered Research and Reviews 2019;6(1):7.

(18) Keenan J, Poland F, Boote J, Howe A, Wythe H, Varley A, et al. ‘We’re passengers sailing in the same ship, but we have our own berths to sleep in’: evaluating patient and public involvement within a regional research programme: an action research project informed by Normalisation Process Theory. PloS one 2019;14(5):e0215953.

(19) Scheffelaar A, Bos N, de Jong M, Triemstra M, van Dulmen S, Luijkx K. Lessons learned from participatory research to enhance client participation in long-term care research: a multiple case study. Research Involvement and Engagement 2020;6(1):1-17.

(20) Stewart MK, Spencer N, Davis AH, Hart C, Boateng B. Developing and piloting a community scientist academy to engage communities and patients in research. Journal of clinical and translational science 2018;2(2):73-78.

(21) Consumer involvement in research (cir): does it work? an evaluation of cancer voices'cir program. Asiapacific journal of clinical oncology: wiley-blackwell 111 river st, hoboken 07030-5774, NJ USA; 2016. hiips://www.cancervoices.org.au/consumer-involveme nt-in-research-program/
