# Supplementary material for: Public and patient involvement: a survey on knowledge, experience and opinions among researchers within a precision oncology European project
Source: BMC Cancer. 2023 Aug 30;23:814. doi: 10.1186/s12885-023-11262-x (PMC10470190; doi:10.1186/s12885-023-11262-x)
Supplement: Supplementary file 2 — Additional file 2. Questionnaire. [file 12885_2023_11262_MOESM2_ESM.docx]

**Additional file 2.** Questionnaire

Public and patient engagement in research (PPI), also called participatory research, is now encouraged by major funding bodies and regulatory Authorities (Gyawali et al. 2019), nevertheless, several barriers exist to its implementation.

We hereby ask you to participate in this survey, which is aimed to assess researchers’ knowledge, experience, attitudes and training needs for participatory PPI. Please note that your participation is entirely voluntary and you are free to decline to participate in this survey. This study has been approved by the Ethical Committee of the Fondazione IRCCS Istituto Nazionale Tumori – Milano (INT 205/21) and will be carried out in 12 European clinical and non-clinical centres. Data will not be anonymously collected, yet responses will be confidentially treated in agreement with the EU General Data Protection Regulation (GDPR) (Regulation EU 2016/679) and results presented only in aggregate form. I give my consent to participate in this web survey:

- I consent
- I do not consent

**°°°°°°°°°°°°°°°°°°°°°°°°°°°°°°°°°°°°°°°°°°°°°°°°°°°°°°°°°°°°°°°°°°°°°°°°°°°°°°°°°°°°°°°°°°°°°°°°°°°°**

**Gender**

- Woman
- Man
- Other
- I prefer not to say

**Year of birth** __________________

**Higher education level**

- Bachelor's or less
- Master's Degree
- PhD
- Other__________________

**Profession**

- Medical Oncologist
- Surgical Oncologist
- Other MD
- Clinical nurse
- Research nurse
- Psychologist
- Basic researcher (biologists, physicists…)
- Epidemiologist
- Statistician
- Bio-informatician
- Economist
- Informatician
- Project manager
- Data manager
- Other __________________

**Primary position**

- Unit/Laboratory/Department Director
- Clinician/researcher
- Trainee (Research fellow, PHD candidate, other student)
- Other _______________________

**Career stage**

- Early-career (≤ 5 years)
- Mid-career (6–15 years)
- Established career (16+ years)

**Primary research setting**

- University
- Hospital/Research hospital
- Other research Institute
- Small and medium-sized enterprise (SME)
- Other _______________________

**Percentage of time spent on research**

- 0%
- 1%-25%
- 26%-50%
- >50%

**°°°°°°°°°°°°°°°°°°°°°°°°°°°°°°°°°°°°°°°°°°°°°°°°°°°°°°°°°°°°°°°°°°°°°°°°°°°°°°°°°°°°°°°°°°°°°°°°°°°°**

**01. Have you ever heard the expression “public and patient involvement in research”, usually abbreviated with PPI?**

- YES
- NO

We acknowledge that there is no unique, universally agreed definition of PPI in health research.

By PPI we mean research being carried out “**with” or “by” members of the** **public** - patients, service users and/or carers, or patient representatives - **rather than “to,” “about” or “for”** them.

Public and patient involvement in research consists in active collaboration about prioritization of research topics, choice of the study design, planning, conducting the study and/or disseminating of research results.

By 'PPI' we do not mean the recruitment of participants for the study (trial, survey, focus groups or interviews), or the dissemination of study results to the patients or public.

**02. Among the following statements, please choose those you are in agreement with. (Select all that apply)**

- PPI is morally/ethically the right thing to do
- Public - patients, service users and/or carers, or patient representatives - can’t contribute in a meaningful way
- PPI should be implemented in any research study
- PPI is not always necessary
- PPI increases the chances of research study success
- I am not convinced of the benefits of PPI
- Other (please specify) ________________________________________________________

**03. Have you ever involved public (patients, service users and/or carers, or patient representatives) in your cancer research activity?**

- Yes
- No

**04. Would you be interested in participating in a PPI training course?**

- Yes
- No

If NO, please specify why____________________________________________________

**05. What do you perceive to be the potential BENEFITS of PPI on cancer research? (Please select up to 3 items)**

- Ensuring that research results are relevant to the public
- Maintaining the connection to the real world
- Improving the research design
- Improving patient recruitment
- Improving dissemination of results
- Improving knowledge and skills of the research team
- Obtaining new perspectives on a research topic
- No benefits
- Other (please specify) ________________________________________________________

**06. What do you perceive to be the potential NEGATIVE EFFECTS of PPI on cancer research? (Please select up to 3 items)**

- Identification of problems not relevant for clinical research
- Involving subjects not representative of target patient population
- Subtracting time/resources/funding from other research activities
- Distrust of researchers/research in general
- Hampering study feasibility
- No negative effects
- Other (please specify) ________________________________________________________

**07.** **What do you perceive to be the BARRIERS to PPI in cancer research?** **(Please select up to 3 items)**

- Patients not understanding the complexity the research topic
- Difficulty in translating scientific to lay language
- Lack of time/resources/funding
- Values/priorities/expectations conflicting among the stakeholders
- Not knowing how to involve representative patients/public
- Lack of patient and public knowledge of research methodology
- Other (please specify) _____________________________________________________

**08. In your opinion, what is REQUIRED for an EFFECTIVE PPI implementation in cancer research? (Please select up to 3 items)**

- A way to measure PPI impact
- PPI dedicated resources (service/function/staff)
- Training for researchers on how to implement PPI
- Training for public and patients
- Methods, materials, information and guidelines for best PPI practice
- PPI endorsement and support by the Institution and leadership
- Financial resources
- Logistical support
- Other (please specify) ________________________________________________________

**09. Which is the priority level of PPI in cancer research given by your institution?**

- High
- Medium
- Low
- Don't know

**10. If PPI was not a requirement of research funders, would you choose to involve patients in your research?**

- Yes
- No
- Don’t know

**11. According to you, how important is it to offer payment to public and patients involved in PPI activities?**

- Very Important
- Important
- Moderately Important
- Slightly Important
- Not Important

**12. According to you, how important is it to recognise the authorship of public and patient in peer-reviewed publications?**

- Very Important
- Important
- Moderately Important
- Slightly Important
- Not Important

**13. How often was PPI included in your cancer research projects in the last 5 years?**

- Always
- Sometimes
- Rarely
- Never
- Don’t know

**14. Number of your current and past cancer research projects in the last 5 years including PPI? ___________________________________**

**15. Please describe your level of satisfaction in applying PPI in your cancer research projects.**

- Very satisfied
- Satisfied
- Neutral
- Dissatisfied
- Very dissatisfied
- Don't know

**16. Why was PPI included in your cancer research?**

**(Please select up to 3 items)**

- It is required by funder(s)
- It is our institutional policy
- It is morally/ethically the right thing to do
- It results in better research
- To improve recruitment and retention of participants
- Don’t know
- Other (please specify) ______________________________________________________

**17. In which activities/phases of the research process have you involved patients or their carers, service users, members of the public? (Select all that apply)**

- Establishing research priorities
- Protocol development (study design, measurements choice, outcome definition)
- Contributing to funding application
- Discussing research methods
- Contributing in writing/reviewing patients information sheets
- Undertaking the research (e.g. identifying or promoting study participation, collecting data….)
- Data analysis and/or interpretation of results
- Dissemination of findings
- Other (please specify) _____________________________________________________

**18. Which kind of PPI participants were involved in your cancer projects? (Select all that apply)**

- Patients and/or potential patients
- Caregivers
- Family members
- Organisations representing patients’ interests (e.g. advocacy organizations)
- Members of the public who are the targets of project
- Other (please specify) _____________________________________________________

**19. What type of training and education have you received to implement PPI?**

**(Please select up to 3 items)**

- I have received no training or education
- I have received informal training (e.g., blogs, website reviews)
- I have received Institutional training (e.g., seminars, courses)
- One-on-one advice and training from a colleague with PPI experience
- One-on-one advice and training from a patient representative with PPI experience
- Informal self-training
- Other (please specify) _____________________________________________________

**20. Which kind of training would be useful to you? (Select all that apply)**

- Case study discussion
- Practical tools and suggestions
- Theoretical bases
- Overview of methods available
- Collection of experiences
- Other (please specify) _____________________________________________________
