# Supplementary figures and images for: Public and patient involvement: a survey on knowledge, experience and opinions among researchers within a precision oncology European project
Source: BMC Cancer. 2023 Aug 30;23:814. doi: 10.1186/s12885-023-11262-x (PMC10470190; doi:10.1186/s12885-023-11262-x)

**Additional file 3.** Participants’ flowchart


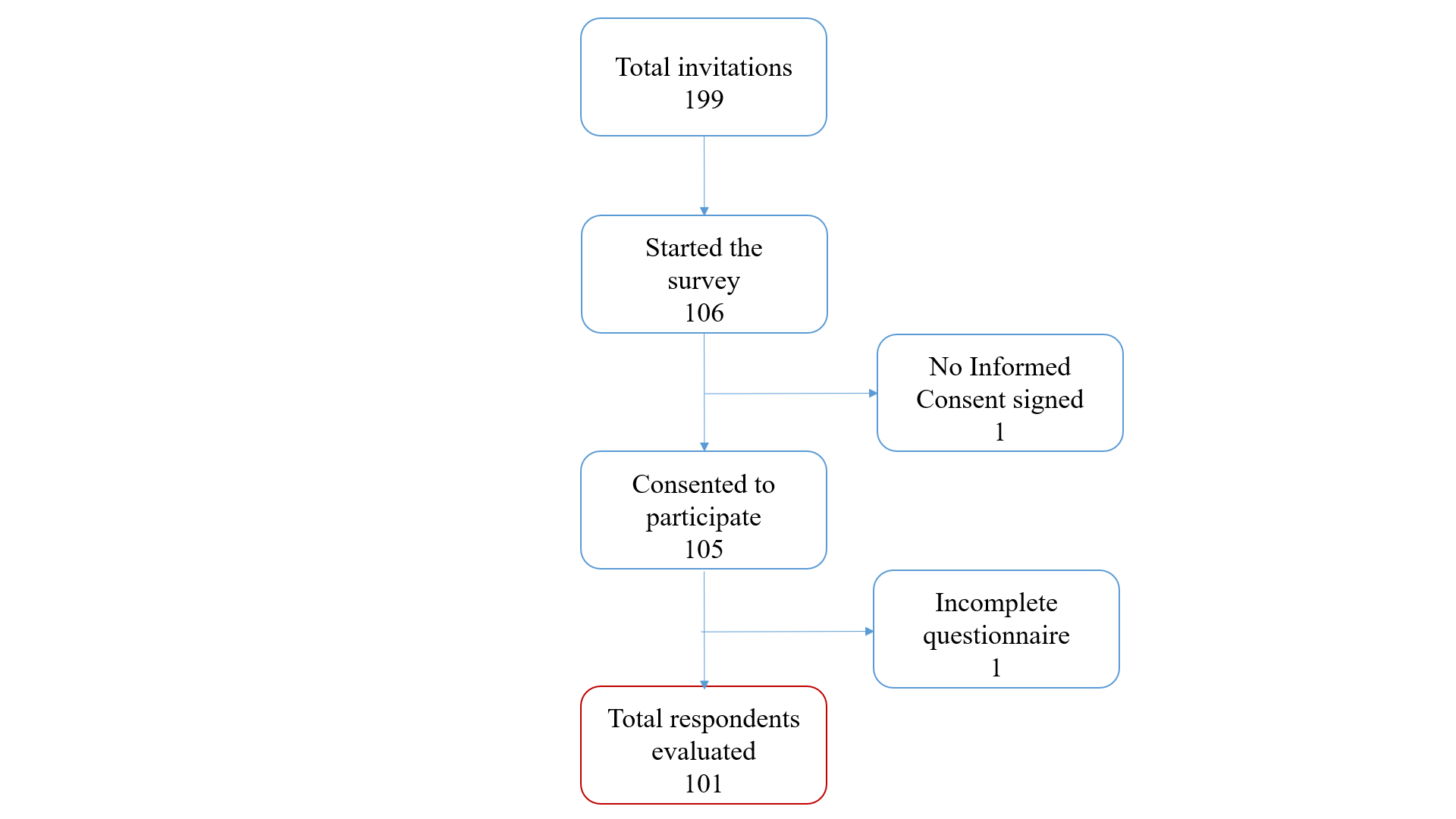

Supplement: Supplementary file 3 — Additional file 3. Participants’ flowchart. [file 12885_2023_11262_MOESM3_ESM.docx]
